# The impact of a bariatric rehabilitation service on weight loss and psychological adjustment - study protocol

**DOI:** 10.1186/1471-2458-12-275

**Published:** 2012-04-05

**Authors:** Amelia Hollywood, Jane Ogden, Christopher Pring

**Affiliations:** 1 Department of Psychology, University of Surrey, Guildford, UK; 2 St Richards Hospital, Spitalfield Lane, Chichester, West Sussex PO19 6SE, UK; 3 Department of Psychology, University of Surrey, Guildford, Surrey GU2 7XH, UK

**Keywords:** Obesity, Surgery, Bariatric, Quality of life, Coping

## Abstract

**Background:**

Bariatric surgery is currently the most effective form of obesity management for those whose BMI is greater than 40 (or 35 with co morbidities). A minority of patients, however, either do not show the desired loss of excess weight or show weight regain by follow up. Research highlights some of the reasons for this variability, most of which centres on the absence of any psychological support with patients describing how although surgery fixes their body, psychological issues relating to dietary control, self esteem, coping and emotional eating remain neglected.

The present study aims to evaluate the impact of a health psychology led bariatric rehabilitation service (BRS) on patient health outcomes. The bariatric rehabilitation service will provide information, support and mentoring pre and post surgery and will address psychological issues such as dietary control, self esteem, coping and emotional eating. The package reflects the rehabilitation services now common place for patients post heart attack and stroke which have been shown to improve patient health outcomes.

**Methods/Design:**

The study is a randomised control trial and patients will be allocated to receive either usual care or the bariatric rehabilitation service pre and post bariatric surgery. Follow up measures of weight loss and psychological issues will be taken at baseline (2 weeks preoperatively), 3, 6 and 12 months postoperatively. The contents of the bariatric service and the follow up measures are based on previous pilot work and have been developed further by the research team working closely with two patient support groups (BOSPA & WLSinfo). This study will take place in St Richard's Hospital in Chichester in the UK.

**Discussion:**

It is predicted that a bariatric rehabilitation service will improve weight loss following surgery and will also facilitate changes in other psychological variables such as quality of life, dietary control, self esteem, coping and emotional eating. This also has cost implications for the NHS and other healthcare providers as improved effectiveness of bariatric surgery reduces the health costs of obese patients in the longer term.

**Trial registration:**

ClinicalTrials.gov NCT01264120.

## Background

Obesity is caused by people consuming more energy than they expend and is associated with reduced life expectancy and many serious conditions including heart disease, stroke, diabetes, cancer, gallstones, fatty liver disease and sleep apnoea [1]. Currently almost two-thirds of UK adults are either overweight or obese with overall costs to society forecast to reach £50 billion per year by 2050 on current trends [2].

Although the most common form of management emphasizes changes in diet and exercise, research indicates that weight loss surgery (wls) is the most effective intervention for weight loss for those whose BMI is greater than 40 (or 35 for those with significant health problems) [3]. A systematic review of wls [4] concluded that the mean percentage excess weight loss (EWL) for the Roux-en-Y gastric bypass was 67% and for the gastric band was 42% at one year. WLS has also been shown to result in improvements in a number of other patient outcomes including quality of life, mood, subjective health status and perceptions of eating control [5,6]. Weight loss outcomes are not consistent for all patients and a large minority of patients either do not show the desired loss of excess weight or regain weight by follow up.

Previous qualitative and quantitative research by the principal investigator has explored the mechanisms involved in successful and failed bariatric surgery to highlight how effectiveness could be improved [6-8]. The results indicated that successful surgery is associated with a reduction in hunger and preoccupation with food and a sense of being more in control of food intake. In contrast, less successful surgery was associated with feeling unprepared for the changes required after surgery, reporting being unsupported in the time following surgery and a sense that although surgery fixes their body, psychological issues relating to dietary control, self esteem, coping and emotional eating remain neglected. This highlights key areas that need to be addressed to improve patient outcomes following surgery. It also confirms the conclusions made by Saltzman et al. [9] who conducted a review of articles related to weight loss surgery and indicated that such patients require multidisciplinary care including psychological input. Furthermore, NICE guidelines [10] state that surgery for obesity should be undertaken only by a multidisciplinary team that can provide psychological support before and after surgery. This is currently missing in the current standard package of care in many centres including for patients at Chichester.

The present study therefore aims to evaluate a health psychology led bariatric rehabilitation service to explore the impact on patient health outcomes following surgery. The bariatric rehabilitation service will offer information, support and mentoring pre and post surgery and finds reflection in the rehabilitation services which are now common place for patients post heart attack and stroke [11]. In particular, research indicates that rehabilitation for other conditions can improve a number of patient outcomes such as return to work, quality of life and health related behaviours [12]. In a similar vein it is predicted that a bariatric rehabilitation service would primarily improve weight loss following surgery but would also facilitate changes in other psychological variables such as dietary control, self esteem, coping and emotional eating.

## Methods/Design

### Study design

The study will involve a trial (open randomised parallel group control trial) with patients allocated to receive either usual care or the bariatric rehabilitation service (BRS) pre and post bariatric (obesity) surgery. Follow up measures will be recorded at 3, 6 and 12 months post-surgery at routine follow-up clinics or by post. In addition, qualitative interviews will be carried out with 20 participants from both arms of the trial (BRS n = 10; usual care n = 10). This study received a favourable ethical opinion from the Kent Research Ethics Committee, National Research Ethics Service.

### Development of the intervention

The content of the BRS and the follow up measures are based on previous qualitative research and pilot work exploring what bariatric patients want and why bariatric surgery either fails or succeeds from the patients' perspective [6-8]. Furthermore, pilot research has explored the impact of bariatric surgery on changes in mood, cognitions and eating behaviours. The quantitative measures and qualitative responses derived from these studies formed the basis of the intervention and the measures for the present study.

### Participants and recruitment

St Richard's Hospital in Chichester, West Sussex, UK, offers a NHS based bariatric service for obese patients with a BMI over 40 (or 35 with serious co morbidities). This is the largest centre in the UK, with approximately 500 patients per year receiving a bariatric operation at this clinic (70% being a primary bypass and 20% bands (the remaining 10% are revisions)). Patients will be recruited if they have been approved for surgery and had their date set for their operation.

Patients will be included if they consent, are aged 18 or over, have attended the bariatric clinic at Chichester, been accepted for surgery and have funding in place (i.e. the PCT has agreed to pay for their surgery). Recruitment will take place over a 12 month period. Those who cannot effectively read or speak English will be excluded as this would pose a difficulty for implementing the intervention and for data collection.

### Randomisation

All patients who fulfil the inclusion criteria will be given an information sheet. They will be given several weeks to read the information and then they will be approached by the researcher, asked if they have any questions regarding the information sheet and if interested offered a written consent form. Once a patient has consented the researcher will reference the third party blinded randomization, which will be provided by the clinical trial unit at Surrey University, to indicate whether they are allocated to either the BRS or usual care.

### Sample size and power calculation

Petrie et al [12] carried out an intervention (34 controls and 31 in the intervention group) with patients post MI which involved the addition of a health psychologist to usual care who focused on a number of psychological factors including beliefs, expectations and behaviour. The results from this study showed that this intervention produced a significant improvement in a certain patient outcome by follow up of about 20% with a standard error of 40%. Based on the information from this study, were the study to be repeated, 85 subjects in each arm would be needed for 90% power for the effect of the intervention on this patient outcome at a two-sided significance level of 0.05. It can be argued that this can be used as the basis for considering the expected improvement for the present study. Buchwald et al [13] reported that the mean percentage of excess weight loss among obese patients offered bariatric surgery was 62%. If the psychological intervention described here improved this weight loss by a further 20% (i.e. to 74%), by the above reasoning, at 0.05 significance 85 patients randomised to each group would give 90% power to demonstrate statistical significance of the intervention. Bands are expected to constitute 20% of all presenting patients. If 90% power is required to find the intervention statistically significant for bands and for bypass, separately, then 85 patients will need to be randomised for each of bands and bypass for each arm of the study. However we hypothesise that bands will be more responsive to the intervention than bypass and hence we will aim for 60 bands and 85 bypass patients in each of the two arms of the study, making a total of 290 patients for the study. Allowing for some attrition and refusals we propose to invite 335 patients to be recruited into the study, ensuring that 180 of these are bands and the remainder bypass. This sample size will enable us to detect a 20% improvement on usual care in the entire sample with power in excess of 90% and will also enable us to carry out some sub group analyses to explore differential effects with 90% power within those patients who receive either a gastric band or a gastric bypass.

### Procedure

Patients attend the bariatric clinic for a multidisciplinary assessment day where they are told if they will be getting the surgery. If they are accepted for surgery they are given an information sheet concerning the present study. Two weeks prior to their operation patients will attend the bariatric clinic for a preoperative appointment where they will have routine tests. At this appointment patients will see the researcher who will explain the trial, obtain informed consent and randomly allocate the patient to either the usual care condition (control group) or the bariatric rehabilitation service (BRS) condition (intervention group). Patients will then complete the baseline questionnaire and either see the health psychologist or not depending on to which condition they are allocated. All patients, from both groups, will complete follow up questionnaires at 3, 6 and 12 months.

### Control group

Those allocated to the usual care (control) group will receive usual management which involves preoperative tests and an information sheet post-operatively informing them about their desired diet. Patients will then return for their surgery after two weeks and after the preoperative tests and after a brief post-surgical stay they will then be discharged. The standard diet sheet they receive takes them through the stages of food progression from only consuming liquids, to soft food then back to all foods. They will return to the clinic at 3 months postoperatively to see the dietician. Patients will also attend routine follow up appointments at 3, 6 and 12 months.

### Intervention group

Those allocated to the BRS (intervention group) will receive usual care as described above plus three 50 minute sessions with a health psychologist pre-operatively (2 weeks prior to surgery), post-operatively (before they are discharged from hospital) and at 3 months follow up. The design of the BRS will be based on the preparation procedures for surgery and cardiac rehabilitation services but will be tailored to the needs of bariatric patients in line with qualitative research carried out by the current author (7; 8). In addition, the BRS has been designed in line with ongoing input from users of two active support groups who have highlighted the need for increased psychological input. The health psychologist will use both didactic methods such as information giving and non didactic methods such as active listening, asking open questions and encouraging reflection. The sessions are fully structured but are flexible to work with the individual patient. In particular the service will address 5 key factors as follows: i) knowledge (ie information about dietary change); ii) beliefs (concerning the causes and solutions to obesity); iii) behaviours (with a focus on diet and physical activity); iv) coping strategies (i.e. managing emotions without using food; identifying alternative and healthy methods of coping; managing other addictions); v) adjustment (i.e. exploring ways to work with the restriction imposed by the operation).

**Figure 1:**
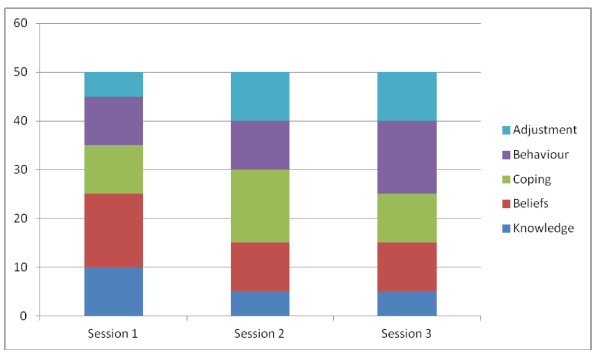
**Suggested focus of each session**.

These key elements will be tailored to each time point for each of the three sessions and the emphasis on each factor will vary according to whether the session is pre surgery, immediately post surgery or at three months follow up. The distribution of these 5 key factors across the three sessions is shown in Figure 1. The health psychologist will be given formal training in order for them to follow the same protocol for each patient in the intervention. They will be given a training manual covering background information, overview of the study protocol, along with a session plan for each of the three sessions. The training manual will be accompanied by guidelines for the focus of each session (see Figure 1) along with details of a recommended script and suggested strategies to be used in each session. In addition, the health psychologist will be asked to provide each patient with a take home message sheet to take away with them after each session which will briefly describe the issues covered in the session and any strategies agreed upon.

### Primary outcome measures

Patients' weight and BMI will be obtained in the clinic to provide the primary endpoint measure of the trial. This will be collected preoperatively a couple of weeks before surgery and postoperatively at 3, 6 and 12 months follow up.

### Secondary outcome measures

Baseline measures of age, sex, weight, height, educational level, and ethnicity will be taken. Patients will also be asked to complete a number of validated measures that have been used previously to assess the impact of bariatric surgery at baseline, 3, 6 and 12 months follow up to evaluate the secondary outcome measures of quality of life, mood, coping and emotional eating (i.e. ADL section of SF-36, [14], profiles of mood states, [15], COPE [16] and DEBQ, [17]).

## Discussion

Obesity is a risk factor for a multitude of illnesses such as heart disease, diabetes and cancer.

If effective, obesity surgery improves a patient's health and reduces their need for NHS care or other healthcare costs. If unsuccessful then the costs include not only subsequent NHS costs due to these other illnesses but also the costs of the unsuccessful operation, the costs of future operation(s) and the emotional cost to the patient. The bariatric rehabilitation service should help to improve the effectiveness of surgery which in the longer term is likely to be cost effective.

This research is a direct response to the needs identified by patients and by offering a more comprehensive bariatric service the success and subsequent health and well being of obese patients should be improved. This research is also in line with the NICE guidelines which promote psychological support both pre and post surgery.

The current service for bariatric patients nationally is very unsystematic. Patients see a surgeon and may be referred to see a clinical psychologist to diagnose any clinical disorders, which might require treatment. All patients then see a dietician. No further psychological support is usually given either before or after surgery. The addition of a health psychologist to their care would bring the bariatric service in line with other clinics such as smoking cessation and rehabilitation post MI and stroke.

If effective then the health psychology led BRS intervention could be easily implemented across the NHS by using existing health psychologists, appointing new health psychologists or even training other members of the service to deliver a health psychology intervention. The additional psychological support may not only improve the patients' outcomes in terms of weight loss and quality of life, but also reduce the need for reversal or revision surgery. Such additional surgery has financial implications for the NHS and is a cost to the patient who has to undergo the risks of further surgery and recover from another operation.

## Competing interests

The authors declare that they have no competing interests.

## Authors' contributions

AH participated in the conception, design and co-ordination of the study, and drafted the manuscript. JO participated in the conception and design of the study, and revised the manuscript. CP participated in the design of the study and reviewed the manuscript. All authors read and approved the final manuscript.

## Pre-publication history

The pre-publication history for this paper can be accessed here:

http://www.biomedcentral.com/1471-2458/12/275/prepub
